# Estrogen for the Treatment of Low Bone Mineral Density in Anorexia Nervosa

**DOI:** 10.20900/jpbs.20220004

**Published:** 2022-07-04

**Authors:** Subhanudh Thavaraputta, Pouneh K. Fazeli

**Affiliations:** Neuroendocrinology Unit, Division of Endocrinology and Metabolism, University of Pittsburgh School of Medicine, Pittsburgh, PA 15213, USA

**Keywords:** anorexia nervosa, bone mineral density, estrogen

## Abstract

Anorexia nervosa is a disorder of chronic, self-induced negative energy balance which typically results in a low body weight. Functional hypothalamic amenorrhea is an adaptive response to states of negative energy balance and chronic undernutrition. A majority of women with anorexia nervosa are amenorrheic with resultant hypoestrogenemia, and longer durations of amenorrhea are associated with lower bone mineral density in this population. In this review, we highlight studies that have investigated the effects of estrogen replacement on bone mineral density in anorexia nervosa, including prospective and randomized studies that show no benefit to treatment with oral estrogen with respect to bone mineral density in either adolescent girls or women with anorexia nervosa. We also review data from a randomized, placebo-controlled study in adolescent girls and a prospective, open-label pilot study in women with anorexia nervosa suggesting that transdermal estrogen may have beneficial effects with respect to bone mineral density in this population.

## FUNCTIONAL HYPOTHALAMIC AMENORRHEA IN ANOREXIA NERVOSA

One key hormonal response to states of undernutrition is the directing of energy away from the reproductive axis [1]. Although reproduction, an energetically costly process, is necessary for the survival of a species, it is not necessary for the survival of an individual, and therefore decreasing energy expenditure on reproduction is advantageous in states of undernutrition [1]. This directing of energy away from the reproductive axis during periods of decreased caloric intake is termed functional hypothalamic amenorrhea or hypogonadotropic hypogonadism.

Anorexia nervosa is a psychiatric disorder characterized by a state of self-induced negative energy balance and typically results in a low body weight due to inappropriately low caloric intake [2]. A majority of women with anorexia nervosa have functional hypothalamic amenorrhea and amenorrhea was previously included in the diagnostic criteria for anorexia nervosa [3]. Functional hypothalamic amenorrhea, as well as the other adaptive responses to chronic undernutrition, which include growth hormone resistance and hypercortisolemia, likely contribute to the negative consequences of long-term undernutrition [1].

One of the most common negative consequences of chronic undernutrition is low bone mineral density [4]. Approximately 35% of women with anorexia nervosa have a bone mineral density value that is more than 2.5 standard deviations lower than the mean of women of similar age and an additional 50% of women have a bone mineral density value more than 1 standard deviation lower than the mean [4]; individuals with anorexia nervosa also have a higher risk of fracture [5-8]. In fact, a prospective study showed that women with anorexia nervosa were seven-times more likely to sustain a non-vertebral fracture compared to women of similar age [9]. Since bone mass accrual occurs during adolescence and peak bone mass is typically achieved before the age of 30 years old [10], a disorder such as anorexia nervosa, which most commonly develops during adolescence [11], can have lifelong effects due to the failure to achieve peak bone mass [12]. This coupled with the fact that anorexia nervosa is a chronic disorder with a long-term recovery rate of only approximately 50–60% [13,14] contribute to a long-term increased risk of fracture in this population [15].

Functional hypothalamic amenorrhea is a result of disruption in hypothalamic secretion of gonadotropin releasing hormone [16-20]. Functional hypothalamic amenorrhea is characterized by low estrogen levels and low estrogen levels increase bone resorption resulting in a loss of bone mass [21-24]. In anorexia nervosa, the length of time with amenorrhea is directly associated with decreased bone mineral density, such that women with longer durations of amenorrhea have lower bone mineral density [12,25].

## ESTROGEN REPLACEMENT IN ANOREXIA NERVOSA

Given the fact that low estrogen levels are common in anorexia nervosa, due to functional hypothalamic amenorrhea, and hypoestrogenemia is associated with higher rates of bone resorption, it is not surprising that estrogen has been investigated as a possible treatment for the low bone mass in anorexia nervosa (Table 1). Three cross-sectional studies investigating the effects of prior oral estrogen use on bone mineral density in anorexia nervosa showed that women with a history of prior oral estrogen use had higher bone mineral density at the lumbar spine compared to women without a prior history of use [12,26,27]. Yet prospective, randomized studies of oral estrogen have not shown the same benefit [28-30]. Multiple prospective studies in both adult and adolescent populations, ranging in length from 9 months to two years, demonstrate that oral estrogen does not result in improvements in bone mineral density in girls or women with anorexia nervosa (Table 1).

## WHAT IS THE POSSIBLE REASON FOR THIS LACK OF BENEFIT OF ORAL ESTROGEN?

Estrogen suppresses IGF-1 production in the liver [36,37]. IGF-1 is a nutritionally-dependent hormone with bone anabolic effects and levels of IGF-1 are decreased in states of starvation due to growth hormone resistance—an energy-preserving adaption in states of undernutrition [38]. IGF-1 is suppressed by estrogen in a route-dependent manner such that oral estrogen suppresses IGF-1 to a greater degree than transdermal estrogen [36]. Therefore, further suppression of IGF-1—a bone anabolic hormone—in anorexia nervosa by oral estrogen may result in the lack of benefit with respect to bone mineral density observed in prospective studies of oral estrogen treatment.

In contrast to oral estrogen, transdermal estrogen leads to an increase in bone mineral density in adolescents with anorexia nervosa [34]. An 18-month randomized placebo-controlled study in adolescent girls with anorexia nervosa demonstrated that girls (ages 12–18 years) who were treated predominantly with transdermal physiologic estrogen had a significantly greater increase (2.6%) in lumbar spine bone mineral density after 18 months as compared to adolescent girls treated with placebo, who had a 0.3% increase in lumbar spine bone mineral density after 18 months [34]. The doses of estrogen that were used in this study were also more physiologic than the more potent formulations used in oral contraceptive pills and importantly IGF-1 suppression is not only dependent on the route of administration, but IGF-1 is also suppressed in a dose-dependent manner, such that lower doses of estradiol are less suppressive than higher doses [37]. Therefore, this randomized, placebo-controlled study demonstrated that physiologic estradiol treatment has a beneficial effect on bone mineral density in adolescent girls with anorexia nervosa.

## CAN WE EXTRAPOLATE THESE DATA TO ADULTS?

Healthy adolescent girls are in a state of bone acquisition whereas by the age of approximately 20–30 years, the majority of bone mass has been accrued and peak bone mass has essentially been achieved [10]. Therefore, bone modeling and remodeling differ in adolescents as compared to adults. When compared to healthy adolescent girls, adolescents with anorexia have a lower bone formation rate but similar bone resorption rate [39-41]. In contrast, compared to normal-weight adult women, women with anorexia nervosa have a lower bone formation rate and a greater degree of bone resorption [42-45]. These differences in bone modeling and remodeling in adults versus adolescents likely contributes to the differences that have been observed in response to treatments for low bone mineral density in adolescents with anorexia nervosa as compared to adults [1]. For example, bisphosphonate treatment has been shown to have a beneficial effect with respect to bone mineral density in adult women with anorexia nervosa [46] but not in adolescents with anorexia nervosa [47]. Similarly, treatment with recombinant human IGF-1 has beneficial effects on bone mineral density in adult women with anorexia nervosa [29] but not adolescent girls [48]. Therefore, although physiologic transdermal estrogen therapy improves bone mineral density in adolescents with anorexia nervosa, it also needs to be studied separately in adult women with anorexia nervosa. We conducted an open-label six-month study in women (*n* = 11) with anorexia nervosa who were a mean (SEM) of 37.2 ± 2.3 years of age [35]. Study participants had anorexia nervosa for a median (interquartile range) of 16 (10, 23) years and were amenorrheic for a median of 157 (36, 180) months [35]. Six months of treatment with a transdermal estradiol patch (45 mcg/24 h) resulted in a mean 2% increase in lumbar spine bone mineral density [35]. A significant change in total hip or femoral neck bone mineral density was not observed after six-months of treatment [35]. As all individuals with an intact uterus must be treated with progesterone in addition to estrogen to prevent endometrial hyperplasia, the patch administered in the study (Climara Pro; Bayer Pharmaceuticals, Whippany, NJ, USA) also included levonorgestrel (0.015 mg/day) [35]. Importantly, administration of continuous estrogen/progesterone typically does not result in cyclic bleeding resembling a menstrual period, which is in contrast to oral estrogen in the form of an oral contraceptive pill. Therefore, oral contraceptives may have the added negative effect of masking amenorrhea, as women with anorexia nervosa may have regular, monthly withdrawal bleeds while using oral contraceptives. In turn, this may hinder recovery, as women using oral contraceptives will not know if they are persistently amenorrheic. Given these promising pilot data suggesting benefit with respect to bone mineral density in adult women with anorexia nervosa, we are now studying the effects of this dose of transdermal estradiol in an 18-month randomized, placebo-controlled study.

## CONCLUSIONS

Although amenorrhea is no longer a part of the DSM criteria for anorexia nervosa, a majority of women with anorexia nervosa have functional hypothalamic amenorrhea and resultant hypoestrogenemia [2,3]. Although duration of amenorrhea is associated with bone mineral density, such that the longer the duration of amenorrhea the lower the bone mineral density in women with anorexia nervosa [12,25], prospective studies investigating the effects of oral estrogen use, predominantly in the form of an oral contraceptive pill, on bone mineral density have not demonstrated benefit. In contrast, physiologic, transdermal estrogen use has been shown to be beneficial with respect to bone mineral density in adolescent girls with anorexia nervosa. As data from adolescent studies cannot be extrapolated to adults due to differences in bone modeling and remodeling in the two populations, the effects of physiologic, transdermal estrogen must also be studied in adults [1]. Preliminary, open-label data in women with anorexia nervosa suggest that transdermal estrogen may also be beneficial in this population [35] and a randomized, placebo-controlled study is currently underway investigating its effects on bone mineral density in women with anorexia nervosa.

## Figures and Tables

**Table 1.** Studies investigating the effects of estrogen on bone mineral density in anorexia nervosa. OCP: oral contraceptive pill; SD: standard deviation; SEM: standard error of the mean; rhIGF-1: recombinant human IGF-1; N/A: not applicable; BMD: bone mineral density.

Study	Study design	Anorexia nervosa study population characteristics	Estrogen type	Follow-up time	Findings
Seeman et al., 1992 [12]	Cross-sectional	65 women with anorexia nervosa (16 women with history of OCP use) Mean age of women with history of OCP use: 27.6 ± 1.9 years	OCP	N/A	Mean BMD at lumbar spine greater in women with history of OCP use as compared to those without history of OCP use
Klibanski et al., 1995 [28]	Randomized	48 study participants with anorexia nervosa (22 randomized to oral estrogen) Age range: 16.3–42.5 years with mean age: 24.9 ± 6.9 years (SD)	Oral estrogen	Mean: 1.5 years	No difference in change in spine BMD in oral estrogen versus placebo overall. In women with baseline ideal body weight of <70%, there was a significant treatment effect: 4% ± 8.9% increase in spine BMD in estrogen group versus 20.1% ± 16.2% decrease in control group (posthoc analysis)
Karlsson et al., 2000 [26]	Cross-sectional	135 women with active anorexia nervosa (58 women with history of estrogen use for a mean of 4.3 years) Mean age of women with history of estrogen use: 28.4 ± 1 (SEM) years Mean age of women without history of estrogen use: 25.9 ± 0.8 years	Oral estrogen	N/A	Areal and volumetric BMD of the L3 vertebra significantly greater in women with history of estrogen use as compared to those without
Grinspoon et al., 2002 [29]	Randomized study of OCP and rhIGF-1	60 women with anorexia nervosa (15 randomized to placebo, 15 randomized to OCP alone, 16 randomized to rhIGF-1 alone, and 14 randomized to OCP+rhIGF-1) Mean age: 25.2 ± 0.7 years	OCP	9 months	No difference in change in lumbar spine BMD when comparing women randomized to OCP to women randomized to no OCP treatment
Golden et al., 2002 [31]	Prospective, observational	50 girls/women (13–21 years of age) with anorexia nervosa (22 received estrogen) Mean age: 16.8 ± 2.3 years (SD)	OCP	Mean: 23.1 months	No difference in change in lumbar spine or femoral neck BMD in estrogen group compared to group not prescribed estrogen after 1 year of follow-up.
Munoz et al., 2002 [32]	Prospective, open-label	38 girls/women with anorexia nervosa (mean age: 17.3 years)	Oral estrogen	1 year	No significant change in lumbar spine BMD after 1 year of treatment
Strokosch et al., 2006 [30]	Randomized, placebo-controlled	43 girls with anorexia nervosa were included in a study of 112 adolescent girls (age: 11–17 years) with either anorexia nervosa or an eating disorder not otherwise specified (EDNOS); 18 with anorexia nervosa randomized to estrogen	OCP	1 year	No difference in change in lumbar spine BMD after 1 year in subset of patients with anorexia nervosa
Legroux-Gerot et al., 2008 [33]	Prospective observational	45 girls/women with anorexia nervosa [age range: 15–41 years with mean (SD) age: 25.3 ± 6.7 years]. Those with a T-score <−2.5 (*n* = 12) were treated with estrogen	Estradiol gel	2 years	No difference in change in lumbar spine or femoral neck BMD in estrogen-treated patients
Misra et al., 2011 [34]	Randomized, placebo-controlled	110 adolescents with anorexia nervosa (ages: 12–18 years with mean age: 16.5 years); 55 randomized to estrogen	Predominantly transdermal estradiol (100 mcg/day); 96 participants randomized to transdermal estradiol or placebo and remaining 14 (bone age <15 years) randomized to oral estrogen or placebo	18 months	Significant 2.6% increase in lumbar spine BMD after 18 months of treatment in estrogen treated group compared to 0.3% in the group not treated with estrogen
Resulaj et al., 2020 [35]	Prospective, open-label	11 women with anorexia nervosa Mean age: 37.2 ± 2.3 years (SEM)	Transdermal estradiol (45 mcg/day)	6 months	2% increase in lumbar spine BMD
Maimoun et al., 2019 [27]	Cross-sectional	305 adolescent and adult women with anorexia nervosa (age range: 14.5–34.9 years with mean age of 99 women with history of OCP use: 22.4 ± 4.6 years (SD)	OCP (84 participants using estrogen-containing OCP and 15 progestin only)	N/A	Areal BMD at lumbar spine and femoral neck greater in women with history of OCP use as compared to those without history of OCP use

## References

[1] Fazeli PK. Low bone mineral density in anorexia nervosa: Treatments and challenges. Clin Rev Bone Miner Metab. 2019;17(2):65–76. 31938025 10.1007/s12018-019-09260-4 PMC6959847

[2] American Psychiatric Association. Diagnostic and statistical manual of mental disorders (DSM-5). Washington (DC, US): American Psychiatric Association; 2013.

[3] American Psychiatric Association. Diagnostic and statistical manual of mental disorders (DSM-IV). 4th ed. Washington (DC, US): American Psychiatric Association; 1994.

[4] Miller KK, Grinspoon SK, Ciampa J, Hier J, Herzog D, Klibanski A. Medical findings in outpatients with anorexia nervosa. Arch Intern Med. 2005;165(5):561–6. 15767533 10.1001/archinte.165.5.561

[5] Vestergaard P, Emborg C, Stoving RK, Hagen C, Mosekilde L, Brixen K. Fractures in patients with anorexia nervosa, bulimia nervosa, and other eating disorders--a nationwide register study. Int J Eat Disord. 2002;32(3):301–8. 12210644 10.1002/eat.10101

[6] Faje AT, Fazeli PK, Miller KK, Katzman DK, Ebrahimi S, Lee H, Fracture risk and areal bone mineral density in adolescent females with anorexia nervosa. Int J Eat Disord. 2014;47(5):458–66. 24430890 10.1002/eat.22248 PMC4053520

[7] Nagata JM, Golden NH, Leonard MB, Copelovitch L, Denburg MR. Assessment of Sex Differences in Fracture Risk Among Patients With Anorexia Nervosa: A Population-Based Cohort Study Using The Health Improvement Network. J Bone Miner Res. 2017;32(5):1082–9. 28019700 10.1002/jbmr.3068 PMC5413380

[8] Frølich J, Winkler LA, Abrahamsen B, Bilenberg N, Hermann AP, Støving RK. Fractures in women with eating disorders-Incidence, predictive factors, and the impact of disease remission: Cohort study with background population controls. Int J Eat Disord. 2020;53(7):1080–7. 31922277 10.1002/eat.23223

[9] Rigotti NA, Neer RM, Skates SJ, Herzog DB, Nussbaum SR. The clinical course of osteoporosis in anorexia nervosa. A longitudinal study of cortical bone mass. JAMA. 1991;265(9):1133–8. 1995999

[10] Heaney RP, Abrams S, Dawson-Hughes B, Looker A, Marcus R, Matkovic V, Peak bone mass. Osteoporos Int. 2000;11(12):985–1009. 11256898 10.1007/s001980070020

[11] Hudson JI, Hiripi E, Pope HG, Kessler RC. The prevalence and correlates of eating disorders in the National Comorbidity Survey Replication. Biol Psychiatry. 2007;61(3):348–58. 16815322 10.1016/j.biopsych.2006.03.040 PMC1892232

[12] Seeman E, Szmukler GI, Formica C, Tsalamandris C, Mestrovic R. Osteoporosis in anorexia nervosa: the influence of peak bone density, bone loss, oral contraceptive use, and exercise. J Bone Miner Res. 1992;7(12):1467–74. 1481732 10.1002/jbmr.5650071215

[13] Lowe B, Zipfel S, Buchholz C, Dupont Y, Reas DL, Herzog W. Long-term outcome of anorexia nervosa in a prospective 21-year follow-up study. Psychol Med. 2001;31(5):881–90. 11459385 10.1017/s003329170100407x

[14] Eddy KT, Tabri N, Thomas JJ, Murray HB, Keshaviah A, Hastings E, Recovery From Anorexia Nervosa and Bulimia Nervosa at 22-Year Follow-Up. J Clin Psychiatry. 2017;78(2):184–9. 28002660 10.4088/JCP.15m10393 PMC7883487

[15] Lucas AR, Melton LJ III, Crowson CS, O’Fallon WM. Long-term fracture risk among women with anorexia nervosa: a population-based cohort study. Mayo Clin Proc. 1999;74(10):972–7. 10918862 10.4065/74.10.972

[16] Wiegelmann W, Solbach HG. Effects of LH-RH on plasma levels of LH and FSH in anorexia nervosa. Horm Metab Res. 1972;4(5):404. 4566864 10.1055/s-0028-1097104

[17] Mecklenburg RS, Loriaux DL, Thompson RH, Andersen AE, Lipsett MB. Hypothalamic dysfunction in patients with anorexia nervosa. Medicine. 1974;53(2):147–59. 4593749 10.1097/00005792-197403000-00003

[18] Travaglini P, Beck-Peccoz P, Ferrari C, Ambrosi B, Paracchi A, Severgnini A, Some aspects of hypothalamic-pituitary function in patients with anorexia nervosa. Acta Endocrinol. 1976;81(2):252–62. 10.1530/acta.0.0810252 813472

[19] Nillius SJ, Fries H, Wide L. Successful induction of follicular maturation and ovulation by prolonged treatment with LH-releasing hormone in women with anorexia nervosa. Am J Obstet Gynecol. 1975;122(8):921–8. 1098466 10.1016/0002-9378(75)90349-x

[20] Boyar RM, Katz J, Finkelstein JW, Kapen S, Weiner H, Weitzman ED, Anorexia nervosa. Immaturity of the 24-hour luteinizing hormone secretory pattern. N Engl J Med. 1974;291(17):861–5. 4412035 10.1056/NEJM197410242911701

[21] Riis BJ, Rodbro P, Christiansen C. The role of serum concentrations of sex steroids and bone turnover in the development and occurrence of postmenopausal osteoporosis. Calcif Tissue Int. 1986;38(6):318–22. 3089552 10.1007/BF02555743

[22] Seeman E. Estrogen, androgen, and the pathogenesis of bone fragility in women and men. Curr Osteoporos Rep. 2004;2(3):90–6. 16036088 10.1007/s11914-004-0016-0

[23] Falahati-Nini A, Riggs BL, Atkinson EJ, O’Fallon WM, Eastell R, Khosla S. Relative contributions of testosterone and estrogen in regulating bone resorption and formation in normal elderly men. J Clin Invest. 2000;106(12):1553–60. 11120762 10.1172/JCI10942 PMC381474

[24] Finkelstein JS, Lee H, Leder BZ, Burnett-Bowie SA, Goldstein DW, Hahn CW, Gonadal steroid-dependent effects on bone turnover and bone mineral density in men. J Clin Invest. 2016;126(3):1114–25. 26901812 10.1172/JCI84137 PMC4767351

[25] Biller BM, Saxe V, Herzog DB, Rosenthal DI, Holzman S, Klibanski A. Mechanisms of osteoporosis in adult and adolescent women with anorexia nervosa. J Clin Endocrinol Metab. 1989;68(3):548–54. 2493036 10.1210/jcem-68-3-548

[26] Karlsson MK, Weigall SJ, Duan Y, Seeman E. Bone size and volumetric density in women with anorexia nervosa receiving estrogen replacement therapy and in women recovered from anorexia nervosa. J Clin Endocrinol Metab. 2000;85(9):3177–82. 10999805 10.1210/jcem.85.9.6796

[27] Maïmoun L, Renard E, Lefebvre P, Bertet H, Philibert P, Seneque M, Oral contraceptives partially protect from bone loss in young women with anorexia nervosa. Fertil Steril. 2019;111(5):1020–9.e2. 30922647 10.1016/j.fertnstert.2019.01.008

[28] Klibanski A, Biller BM, Schoenfeld DA, Herzog DB, Saxe VC. The effects of estrogen administration on trabecular bone loss in young women with anorexia nervosa. J Clin Endocrinol Metab. 1995;80(3):898–904. 7883849 10.1210/jcem.80.3.7883849

[29] Grinspoon S, Thomas L, Miller K, Herzog D, Klibanski A. Effects of recombinant human IGF-I and oral contraceptive administration on bone density in anorexia nervosa. J Clin Endocrinol Metab. 2002;87(6):2883–91. 12050268 10.1210/jcem.87.6.8574

[30] Strokosch GR, Friedman AJ, Wu SC, Kamin M. Effects of an oral contraceptive (norgestimate/ethinyl estradiol) on bone mineral density in adolescent females with anorexia nervosa: a double-blind, placebo-controlled study. J Adolesc Health. 2006;39(6):819–27. 17116511 10.1016/j.jadohealth.2006.09.010

[31] Golden NH, Lanzkowsky L, Schebendach J, Palestro CJ, Jacobson MS, Shenker IR. The effect of estrogen-progestin treatment on bone mineral density in anorexia nervosa. J Pediatr Adolesc Gynecol. 2002;15(3):135–43. 12106749 10.1016/s1083-3188(02)00145-6

[32] Munoz MT, Morande G, Garcia-Centenera JA, Hervas F, Pozo J, Argente J. The effects of estrogen administration on bone mineral density in adolescents with anorexia nervosa. Eur J Endocrinol. 2002;146(1):45–50. 11751066 10.1530/eje.0.1460045

[33] Legroux-Gerot I, Vignau J, Collier F, Cortet B. Factors influencing changes in bone mineral density in patients with anorexia nervosa-related osteoporosis: the effect of hormone replacement therapy. Calcif Tissue Int. 2008;83(5):315–23. 18836675 10.1007/s00223-008-9173-y

[34] Misra M, Katzman D, Miller KK, Mendes N, Snelgrove D, Russell M, Physiologic estrogen replacement increases bone density in adolescent girls with anorexia nervosa. J Bone Miner Res. 2011;26(10):2430–8. 21698665 10.1002/jbmr.447 PMC3304439

[35] Resulaj M, Polineni S, Meenaghan E, Eddy K, Lee H, Fazeli PK. Transdermal Estrogen in Women With Anorexia Nervosa: An Exploratory Pilot Study. JBMR Plus. 2020;4(1):e10251. 31956852 10.1002/jbm4.10251 PMC6957987

[36] Weissberger AJ, Ho KK, Lazarus L. Contrasting effects of oral and transdermal routes of estrogen replacement therapy on 24-hour growth hormone (GH) secretion, insulin-like growth factor I, and GH-binding protein in postmenopausal women. J Clin Endocrinol Metab. 1991;72(2):374–81. 1991807 10.1210/jcem-72-2-374

[37] Kam GY, Leung KC, Baxter RC, Ho KK. Estrogens exert route- and dose-dependent effects on insulin-like growth factor (IGF)-binding protein-3 and the acid-labile subunit of the IGF ternary complex. J Clin Endocrinol Metab. 2000;85(5):1918–22. 10843175 10.1210/jcem.85.5.6527

[38] Fazeli PK, Klibanski A. Determinants of GH resistance in malnutrition. J Endocrinol. 2014;220(3):R57–65. 24363451 10.1530/JOE-13-0477 PMC3994591

[39] Soyka LA, Grinspoon S, Levitsky LL, Herzog DB, Klibanski A. The effects of anorexia nervosa on bone metabolism in female adolescents. J Clin Endocrinol Metab. 1999;84(12):4489–96. 10599707 10.1210/jcem.84.12.6207

[40] Saggese G, Bertelloni S, Baroncelli GI, Di Nero G. Serum levels of carboxyterminal propeptide of type I procollagen in healthy children from 1st year of life to adulthood and in metabolic bone diseases. Eur J Pediatr. 1992;151(10):764–8. 1425800 10.1007/BF01959087

[41] Heer M, Mika C, Grzella I, Heussen N, Herpertz-Dahlmann B. Bone turnover during inpatient nutritional therapy and outpatient follow-up in patients with anorexia nervosa compared with that in healthy control subjects. Am J Clin Nutr. 2004;80(3):774–81. 15321821 10.1093/ajcn/80.3.774

[42] Hotta M, Fukuda I, Sato K, Hizuka N, Shibasaki T, Takano K. The relationship between bone turnover and body weight, serum insulin-like growth factor (IGF) I, and serum IGF-binding protein levels in patients with anorexia nervosa. J Clin Endocrinol Metab. 2000;85(1):200–6. 10634387 10.1210/jcem.85.1.6321

[43] Weinbrenner T, Zittermann A, Gouni-Berthold I, Stehle P, Berthold HK. Body mass index and disease duration are predictors of disturbed bone turnover in anorexia nervosa. A case-control study. Eur J Clin Nutr. 2003;57(10):1262–7. 14506487 10.1038/sj.ejcn.1601683

[44] Bolton JG, Patel S, Lacey JH, White S. A prospective study of changes in bone turnover and bone density associated with regaining weight in women with anorexia nervosa. Osteoporos Int. 2005;16(12):1955–62. 16027954 10.1007/s00198-005-1972-7

[45] Viapiana O, Gatti D, Dalle Grave R, Todesco T, Rossini M, Braga V, Marked increases in bone mineral density and biochemical markers of bone turnover in patients with anorexia nervosa gaining weight. Bone. 2007;40(4):1073–7. 17240212 10.1016/j.bone.2006.11.015

[46] Miller KK, Meenaghan E, Lawson EA, Misra M, Gleysteen S, Schoenfeld D, Effects of risedronate and low-dose transdermal testosterone on bone mineral density in women with anorexia nervosa: a randomized, placebo-controlled study. J Clin Endocrinol Metab. 2011;96(7):2081–8. 21525157 10.1210/jc.2011-0380 PMC3135194

[47] Golden NH, Iglesias EA, Jacobson MS, Carey D, Meyer W, Schebendach J, Alendronate for the treatment of osteopenia in anorexia nervosa: a randomized, double-blind, placebo-controlled trial. J Clin Endocrinol Metab. 2005;90(6):3179–85. 15784715 10.1210/jc.2004-1659

[48] Singhal V, Bose A, Slattery M, Haines MS, Goldstein MA, Gupta N, Effect of Transdermal Estradiol and Insulin-like Growth Factor-1 on Bone Endpoints of Young Women With Anorexia Nervosa. J Clin Endocrinol Metab. 2021;106(7):2021–35. 33693703 10.1210/clinem/dgab145 PMC8427708

